# (1*S*,2*R*)-1-[(*E*)-(Thio­phen-2-yl­methyl­idene)amino]­indan-2-ol

**DOI:** 10.1107/S1600536812032783

**Published:** 2012-07-28

**Authors:** Min Jeong Go, Ka Hyun Park, Hwi Hyun Lee, Junseong Lee

**Affiliations:** a Department of Chemistry, Chonnam National University, Gwangju 500-757, Republic of Korea

## Abstract

In the title compound, C_14_H_13_NOS, the dihedral angle formed by the mean planes through the indane ring system and the thio­phene ring is 85.04 (11)°. The imine bond is located in the thio­phene plane [the S—C—C—N torsion angle is 0.00 (3)°] and an intra­molecular O—H⋯N hydrogen bond is observed.

## Related literature

For metal complexes containing amino­indanol ligands, see: Lee *et al.* (2007 ▶); Flores-Lopes *et al.* (2000 ▶). For metal comlexes with thio­phene-type ligands, see: Jeong *et al.* (2011 ▶); Dong *et al.* (2006 ▶); Lee *et al.* (1999 ▶).
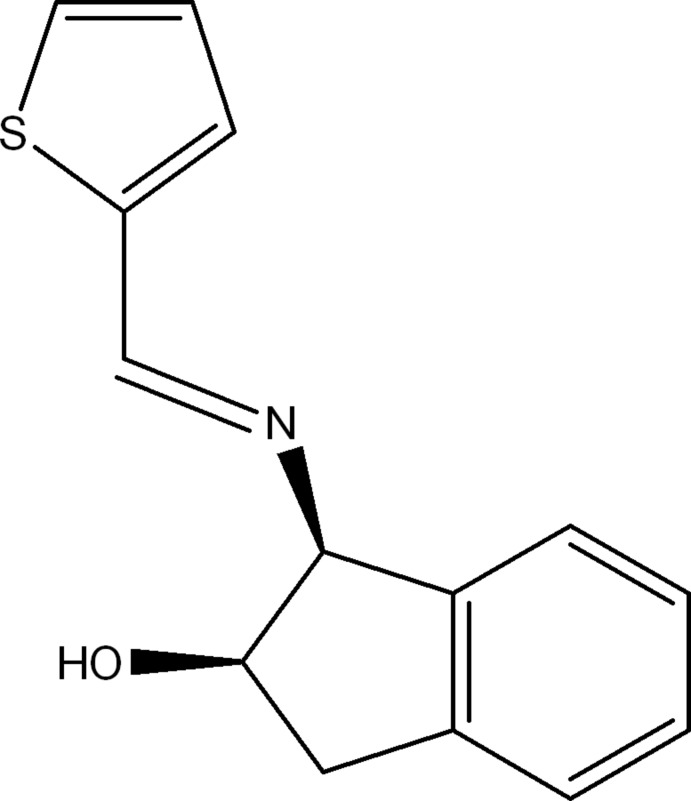


## Experimental

### 

#### Crystal data

C_14_H_13_NOS
*M* *_r_* = 243.31
Monoclinic, 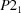

*a* = 5.8640 (2) Å
*b* = 13.4454 (5) Å
*c* = 8.0118 (3) Å
β = 92.258 (2)°
*V* = 631.19 (4) Å^3^
*Z* = 2
Mo *K*α radiation
μ = 0.24 mm^−1^
*T* = 296 K
0.25 × 0.20 × 0.15 mm

#### Data collection

Bruker APEXII CCD diffractometer
Absorption correction: multi-scan (*SADABS*; Bruker, 2009 ▶) *T*_min_ = 0.943, *T*_max_ = 0.965
7148 measured reflections
3182 independent reflections
2848 reflections with *I* > 2σ(*I*)
*R*_int_ = 0.024

#### Refinement

*R*[*F* ^2^ > 2σ(*F* ^2^)] = 0.043
*wR*(*F* ^2^) = 0.131
*S* = 1.03
3182 reflections
155 parameters
1 restraint
H-atom parameters constrained
Δρ_max_ = 0.25 e Å^−3^
Δρ_min_ = −0.27 e Å^−3^
Absolute structure: Flack (1983 ▶), 1201 Friedel pairs
Flack parameter: 0.06 (8)

### 

Data collection: *APEX2* (Bruker, 2009 ▶); cell refinement: *SAINT* (Bruker, 2009 ▶); data reduction: *SAINT*; program(s) used to solve structure: *SHELXS97* (Sheldrick, 2008 ▶); program(s) used to refine structure: *SHELXL97* (Sheldrick, 2008 ▶); molecular graphics: *SHELXTL* (Sheldrick, 2008 ▶); software used to prepare material for publication: *SHELXTL*.

## Supplementary Material

Crystal structure: contains datablock(s) I, global. DOI: 10.1107/S1600536812032783/ff2077sup1.cif

Structure factors: contains datablock(s) I. DOI: 10.1107/S1600536812032783/ff2077Isup2.hkl

Supplementary material file. DOI: 10.1107/S1600536812032783/ff2077Isup3.cdx

Supplementary material file. DOI: 10.1107/S1600536812032783/ff2077Isup4.cml

Additional supplementary materials:  crystallographic information; 3D view; checkCIF report

## Figures and Tables

**Table 1:** Hydrogen-bond geometry (Å, °)

*D*—H⋯*A*	*D*—H	H⋯*A*	*D*⋯*A*	*D*—H⋯*A*
O—H1⋯N	0.82	2.22	2.682 (3)	116

**Fig. 1.**
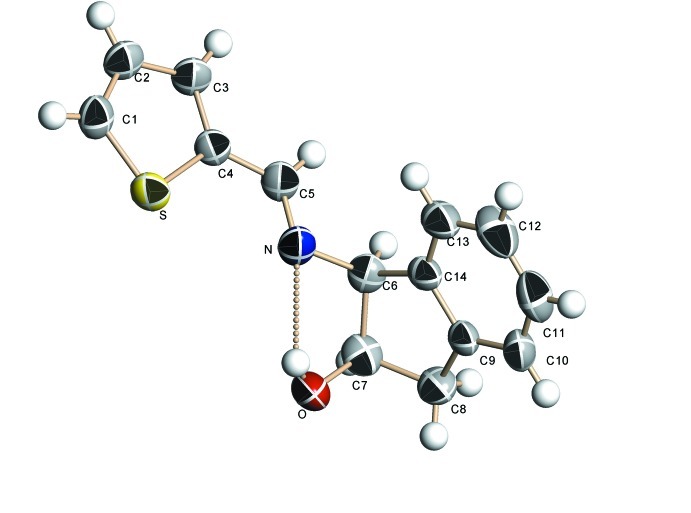
Molecular structure of the title compound, with the atom-numbering scheme. Displacement ellipsoids are drawn at the 50% probability level. H atoms are presented as spheres of arbitrary radius. Hydrogen bonds are drawn as dashed lines.

## supplementary crystallographic information

### Comment

Multidentate ligands having thiophene groups have received a great attention due to their unique binding abilities (Jeong *et al.*, 2011; Dong *et al.*, 2006; Lee *et al.*, 1999). Aminoindaol type ligands have been also extensively used as chiral chelating ligands (Lee *et al.*, 2007; Flores-Lopes *et al.*, 2000). As a part of our ongoing project on the synthesis of new ONS-type tridentate monoanionic chelating ligands, the title compound was synthesized by the reaction of 2-thiophene carboxaldehyde with (1*S*,2*R*)-(-)-*cis*-amino-2-indanol and its crystal structure is reported herein.

In the title compound (Fig. 1), the C7 carbon atom is displaced by 0.450 (2) Å from the mean plane defined by the C6/C8–C14 atoms of the indane ring system. The dihedral angle formed by the mean planes through the indane ring system and the thiophene is 85.04 (11) °. The molecular conformation is stabilized by an intramolecular O—H···N hydrogen bond (Table 1). The absolute configuration is assigned on the basis of the Flack parameter, which is consistent with the known configuration of the indanol employed in the synthesis.

### Experimental

A mixture of (1*S*,2*R*)-(-)-amino-2-indanol (0.149 g,1 mmol) and 2-thiophene carboxaldehyde (0.112 g,1 mmol) was stirred in ethanol for 24 h. The residue, obtained by removing the solvent under vacuum, was recrystallized in dichloromethane. The desired product was isolated as white crystals after the solution remained at -20 °C in a refrigerator for a few days (yield 80%, 0.195 g).

### Refinement

The H-atoms were included in calculated positions and treated as riding atoms: *U*_iso_(H) = 1.2Ueq(parent C-atom),*U*_iso_(H) = 1.5Ueq(parent O-atom).

### Crystal data

**Table:**

C_14_H_13_NOS	*F*(000) = 256
*M**_r_* = 243.31	*D*_x_ = 1.280 Mg m^−^^3^
Monoclinic, *P*2_1_	Mo *K*α radiation, λ = 0.71073 Å
Hall symbol: P 2yb	Cell parameters from 5424 reflections
*a* = 5.8640 (2) Å	θ = 2.5–30.4°
*b* = 13.4454 (5) Å	µ = 0.24 mm^−^^1^
*c* = 8.0118 (3) Å	*T* = 296 K
β = 92.258 (2)°	Block, white
*V* = 631.19 (4) Å^3^	0.25 × 0.20 × 0.15 mm
*Z* = 2	

### Data collection

**Table:**

Bruker APEXII CCD diffractometer	3182 independent reflections
Radiation source: fine-focus sealed tube	2848 reflections with *I* > 2σ(*I*)
Graphite monochromator	*R*_int_ = 0.024
φ and ω scans	θ_max_ = 30.7°, θ_min_ = 2.5°
Absorption correction: multi-scan (*SADABS*; Bruker, 2009)	*h* = −8→7
*T*_min_ = 0.943, *T*_max_ = 0.965	*k* = −15→18
7148 measured reflections	*l* = −11→11

### Refinement

**Table:**

Refinement on *F*^2^	Secondary atom site location: difference Fourier map
Least-squares matrix: full	Hydrogen site location: inferred from neighbouring sites
*R*[*F*^2^ > 2σ(*F*^2^)] = 0.043	H-atom parameters constrained
*wR*(*F*^2^) = 0.131	*w* = 1/[σ^2^(*F*_o_^2^) + (0.1*P*)^2^] where *P* = (*F*_o_^2^ + 2*F*_c_^2^)/3
*S* = 1.03	(Δ/σ)_max_ < 0.001
3182 reflections	Δρ_max_ = 0.25 e Å^−^^3^
155 parameters	Δρ_min_ = −0.27 e Å^−^^3^
1 restraint	Absolute structure: Flack (1983), 1201 Friedel pairs
Primary atom site location: structure-invariant direct methods	Flack parameter: 0.06 (8)

### Special details

**Table:**

Geometry. All e.s.d.'s (except the e.s.d. in the dihedral angle between two l.s. planes) are estimated using the full covariance matrix. The cell e.s.d.'s are taken into account individually in the estimation of e.s.d.'s in distances, angles and torsion angles; correlations between e.s.d.'s in cell parameters are only used when they are defined by crystal symmetry. An approximate (isotropic) treatment of cell e.s.d.'s is used for estimating e.s.d.'s involving l.s. planes.
Refinement. Refinement of *F*^2^ against ALL reflections. The weighted *R*-factor *wR* and goodness of fit *S* are based on *F*^2^, conventional *R*-factors *R* are based on *F*, with *F* set to zero for negative *F*^2^. The threshold expression of *F*^2^ > σ(*F*^2^) is used only for calculating *R*-factors(gt) *etc*. and is not relevant to the choice of reflections for refinement. *R*-factors based on *F*^2^ are statistically about twice as large as those based on *F*, and *R*- factors based on ALL data will be even larger.

### Fractional atomic coordinates and isotropic or equivalent isotropic displacement parameters (Å^2^)

**Table:**

	*x*	*y*	*z*	*U*_iso_*/*U*_eq_	
O	0.3762 (3)	0.41344 (15)	0.6763 (2)	0.0694 (4)	
H1	0.3962	0.4736	0.6845	0.104*	
S	0.84192 (7)	0.69970 (5)	0.55448 (6)	0.05798 (16)	
C9	0.3596 (3)	0.40337 (14)	1.0537 (2)	0.0437 (4)	
C14	0.5379 (3)	0.46986 (12)	1.0333 (2)	0.0431 (3)	
C4	1.0148 (3)	0.64078 (15)	0.7010 (2)	0.0473 (4)	
C13	0.5668 (4)	0.55083 (16)	1.1369 (3)	0.0602 (5)	
H13	0.6866	0.5950	1.1232	0.072*	
C3	1.2263 (3)	0.68631 (19)	0.7167 (3)	0.0552 (5)	
H3	1.3433	0.6657	0.7904	0.066*	
C1	1.0474 (3)	0.78383 (18)	0.5133 (3)	0.0567 (5)	
H1A	1.0286	0.8349	0.4356	0.068*	
C5	0.9397 (3)	0.55622 (17)	0.7985 (3)	0.0524 (4)	
H5	1.0416	0.5274	0.8761	0.063*	
C6	0.6804 (3)	0.43819 (15)	0.8891 (3)	0.0508 (4)	
H6	0.8190	0.4043	0.9312	0.061*	
C2	1.2422 (3)	0.76835 (17)	0.6062 (3)	0.0573 (5)	
H2	1.3724	0.8073	0.5985	0.069*	
C8	0.3710 (4)	0.32062 (16)	0.9292 (3)	0.0570 (5)	
H8A	0.2204	0.3041	0.8830	0.068*	
H8B	0.4395	0.2616	0.9794	0.068*	
C7	0.5206 (4)	0.36346 (16)	0.7957 (3)	0.0574 (5)	
H7	0.6085	0.3110	0.7428	0.069*	
C12	0.4134 (6)	0.5654 (2)	1.2626 (3)	0.0789 (8)	
H12	0.4314	0.6194	1.3345	0.095*	
C10	0.2064 (4)	0.4177 (2)	1.1781 (3)	0.0594 (5)	
H10	0.0874	0.3732	1.1925	0.071*	
C11	0.2347 (5)	0.5002 (3)	1.2812 (3)	0.0742 (7)	
H11	0.1313	0.5117	1.3641	0.089*	
N	0.7401 (3)	0.52068 (15)	0.7810 (2)	0.0549 (4)	

### Atomic displacement parameters (Å^2^)

**Table:**

	*U*^11^	*U*^22^	*U*^33^	*U*^12^	*U*^13^	*U*^23^
O	0.0800 (10)	0.0776 (11)	0.0494 (8)	−0.0161 (9)	−0.0110 (7)	0.0048 (8)
S	0.0389 (2)	0.0805 (3)	0.0542 (3)	0.0031 (2)	−0.00272 (16)	0.0050 (3)
C9	0.0500 (8)	0.0434 (8)	0.0370 (8)	0.0001 (6)	−0.0051 (6)	0.0072 (6)
C14	0.0453 (8)	0.0416 (8)	0.0418 (8)	0.0025 (6)	−0.0075 (6)	0.0049 (7)
C4	0.0335 (7)	0.0623 (10)	0.0462 (9)	0.0047 (6)	0.0029 (6)	0.0021 (8)
C13	0.0775 (14)	0.0474 (10)	0.0549 (12)	−0.0071 (9)	−0.0102 (10)	0.0007 (9)
C3	0.0331 (6)	0.0706 (13)	0.0619 (11)	0.0030 (8)	0.0003 (6)	0.0081 (10)
C1	0.0487 (9)	0.0717 (12)	0.0505 (11)	0.0071 (9)	0.0133 (8)	0.0074 (9)
C5	0.0376 (7)	0.0647 (12)	0.0548 (10)	0.0089 (7)	0.0015 (7)	0.0091 (9)
C6	0.0395 (8)	0.0552 (10)	0.0576 (11)	0.0098 (7)	0.0010 (7)	0.0069 (8)
C2	0.0385 (8)	0.0706 (13)	0.0634 (12)	−0.0013 (8)	0.0105 (8)	0.0034 (10)
C8	0.0693 (12)	0.0416 (9)	0.0596 (13)	−0.0063 (8)	−0.0029 (9)	0.0023 (8)
C7	0.0661 (12)	0.0512 (10)	0.0552 (11)	0.0080 (8)	0.0043 (9)	−0.0086 (9)
C12	0.125 (2)	0.0626 (15)	0.0490 (13)	0.0113 (14)	−0.0034 (13)	−0.0128 (11)
C10	0.0629 (12)	0.0736 (14)	0.0417 (10)	−0.0035 (9)	0.0018 (8)	0.0130 (9)
C11	0.0851 (17)	0.0953 (19)	0.0427 (11)	0.0132 (14)	0.0091 (10)	0.0013 (12)
N	0.0383 (7)	0.0697 (11)	0.0569 (10)	0.0033 (7)	0.0031 (6)	0.0104 (8)

### Geometric parameters (Å, º)

**Table:**

O—C7	1.421 (3)	C1—H1A	0.9300
O—H1	0.8200	C5—N	1.267 (3)
S—C1	1.695 (2)	C5—H5	0.9300
S—C4	1.7143 (19)	C6—N	1.458 (3)
C9—C10	1.381 (3)	C6—C7	1.546 (3)
C9—C14	1.390 (2)	C6—H6	0.9800
C9—C8	1.498 (3)	C2—H2	0.9300
C14—C13	1.376 (3)	C8—C7	1.523 (3)
C14—C6	1.513 (3)	C8—H8A	0.9700
C4—C3	1.384 (3)	C8—H8B	0.9700
C4—C5	1.457 (3)	C7—H7	0.9800
C13—C12	1.390 (4)	C12—C11	1.379 (5)
C13—H13	0.9300	C12—H12	0.9300
C3—C2	1.420 (3)	C10—C11	1.390 (4)
C3—H3	0.9300	C10—H10	0.9300
C1—C2	1.355 (3)	C11—H11	0.9300
			
C7—O—H1	109.5	C14—C6—H6	110.1
C1—S—C4	92.06 (10)	C7—C6—H6	110.1
C10—C9—C14	120.60 (19)	C1—C2—C3	112.79 (18)
C10—C9—C8	129.15 (19)	C1—C2—H2	123.6
C14—C9—C8	110.23 (16)	C3—C2—H2	123.6
C13—C14—C9	120.86 (19)	C9—C8—C7	103.23 (16)
C13—C14—C6	128.70 (18)	C9—C8—H8A	111.1
C9—C14—C6	110.44 (16)	C7—C8—H8A	111.1
C3—C4—C5	125.80 (18)	C9—C8—H8B	111.1
C3—C4—S	111.18 (15)	C7—C8—H8B	111.1
C5—C4—S	122.99 (14)	H8A—C8—H8B	109.1
C14—C13—C12	118.8 (2)	O—C7—C8	107.87 (19)
C14—C13—H13	120.6	O—C7—C6	110.60 (17)
C12—C13—H13	120.6	C8—C7—C6	105.13 (18)
C4—C3—C2	111.57 (17)	O—C7—H7	111.0
C4—C3—H3	124.2	C8—C7—H7	111.0
C2—C3—H3	124.2	C6—C7—H7	111.0
C2—C1—S	112.40 (17)	C11—C12—C13	120.3 (2)
C2—C1—H1A	123.8	C11—C12—H12	119.8
S—C1—H1A	123.8	C13—C12—H12	119.8
N—C5—C4	122.17 (19)	C9—C10—C11	118.3 (2)
N—C5—H5	118.9	C9—C10—H10	120.9
C4—C5—H5	118.9	C11—C10—H10	120.9
N—C6—C14	113.08 (16)	C12—C11—C10	121.1 (2)
N—C6—C7	111.23 (18)	C12—C11—H11	119.4
C14—C6—C7	102.12 (15)	C10—C11—H11	119.4
N—C6—H6	110.1	C5—N—C6	117.62 (17)

### Hydrogen-bond geometry (Å, º)

**Table:**

*D*—H···*A*	*D*—H	H···*A*	*D*···*A*	*D*—H···*A*
O—H1···N	0.82	2.22	2.682 (3)	116
